# Supplementary material for: A Global Screen for Assembly State Changes of the Mitotic Proteome by SEC-SWATH-MS
Source: Cell Syst. 2020 Feb 26;10(2):133–155.e6. doi: 10.1016/j.cels.2020.01.001 (PMC7042714; doi:10.1016/j.cels.2020.01.001)

Q9Y221 | NIP7_HUMAN | NIP7 CGI−37 HSPC031 HSPC180 OK/SW−cl.76 OK/SW−cl.78
Monomer MW [kDa]: 20.463 Monomer expected elution fraction: 54

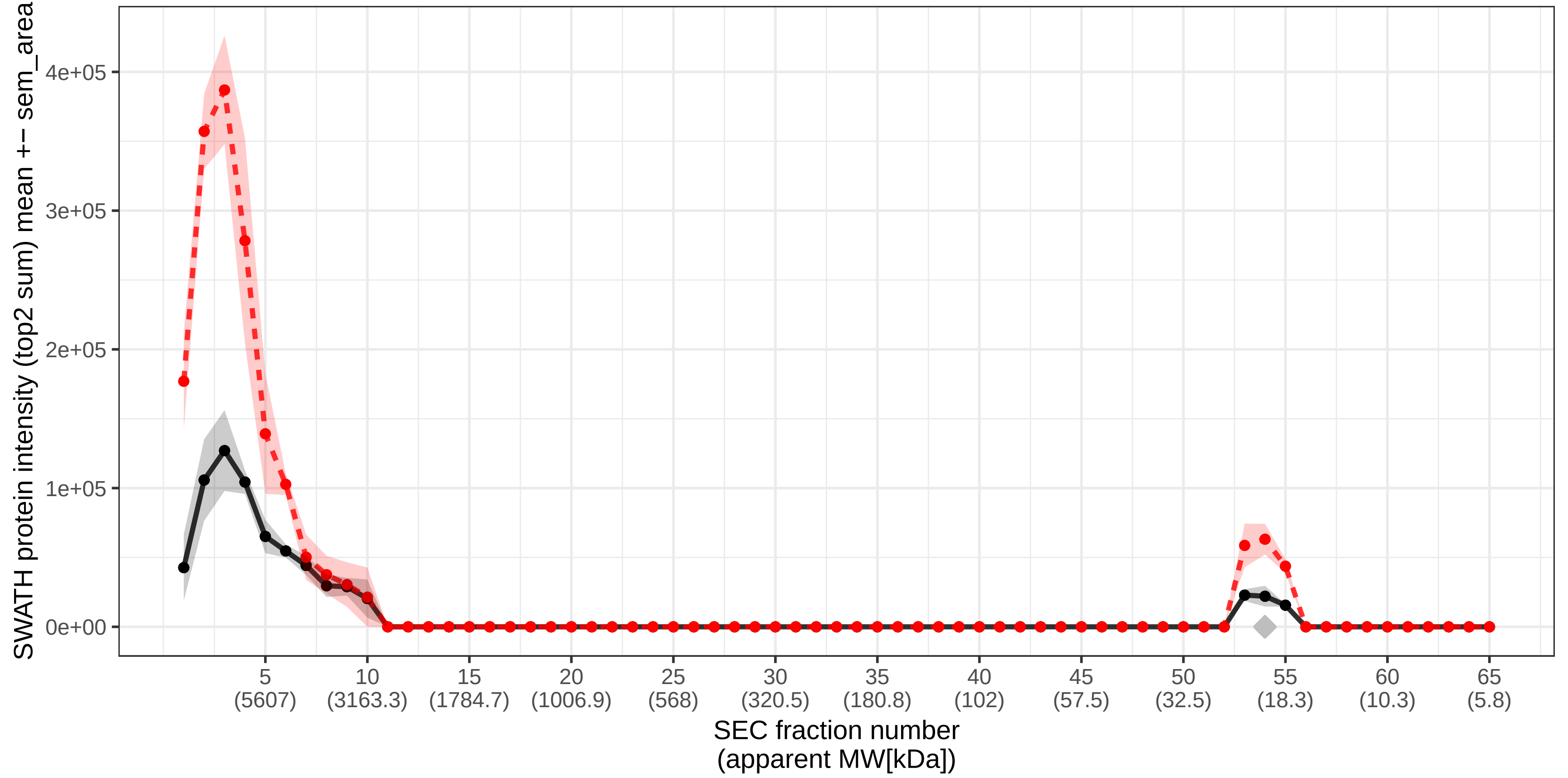

Supplement: Data S1. SEC-SWATH-MS Protein Chromatograms, Related to Figure 1 [file mmc6.zip › SECchrom_Q9Y221_NIP7_HUMAN_NIP7_CGI-37_HSPC031_HSPC180_OK_S.pdf]
